# Lathyrol affects the expression of AR and PSA and inhibits the malignant behavior of RCC cells

**DOI:** 10.1515/med-2024-1136

**Published:** 2025-02-04

**Authors:** Shengyou Song, Lunwei Tai, Lei Zhou, Junling Jiang, Junfeng Zhao

**Affiliations:** The Second Clinical Medical College of Henan University of Traditional Chinese Medicine, Zhengzhou, Henan, 450002, China; Department of Urology, Henan Provincial Hospital of Traditional Chinese Medicine, Zhengzhou, Henan 450002, China

**Keywords:** lathyrol, renal cell cancer, androgen receptor, malignant behavior of tumor cells

## Abstract

**Objective:**

To investigate how lathyrol affects aggressive behaviors and related proteins of the androgen receptor (AR) 786-O cells.

**Methods:**

786-O cells were cultured *in vitro* and divided into these groups at random: the dimethylsulfoxide (DMSO) control group (A group), negative control group (B group), and experimental group (C group). Cells in A group were grown in DMSO working medium (contained RPMI 1640 medium and 1% DMSO), B group cells were cultured in nilutamide working medium (contained DMSO working medium and 325 μg/mL nilutamide), while those in C group were cultured in lathyrol working medium (contained DMSO working medium and 300 μg/mL lathyrol). Cell proliferation was measured via CCK-8 assays, and cell apoptosis was examined through terminal deoxynucleotidyl transferase-mediated dUTP nick end labeling staining. Scratch tests and Transwell invasion tests were used to evaluate cell movement and penetration. The expression information about p-AR, AR, p-Akt, ki67, caspase3, cleaved-caspase3, Bcl-2, Bax, caspase9, cleaved-caspase9, and GAPDH proteins was investigated through western blotting. Immunocytochemistry was used to identify the 786-O cells’ secretion level of matrix metalloproteinase 2 (MMP2), MMP9, and prostate-specific antigen (PSA) proteins.

**Results:**

The negative control and experimental groups’ cells exhibited reduced proliferation, migration, and invasion and increased apoptosis after 24 h treatment. Furthermore, these two group cells exhibited a notable reduction in the status of Ki67, Bcl-2, MMP2, MMP9, and p-Akt (*P* < 0.05) and significantly increased the expressions of AR, p-AR, Bax, cleaved-caspase3, and cleaved-caspase9 (*P* < 0.05). There was no statistical distance in PSA, caspase3, and caspase9 expressions among the three groups (*P* > 0.05).

**Conclusion:**

*In vitro,* lathyrol and nilutamide exert notable anticancer effects by effectively suppressing the proliferation, migration, and invasion of 786-O cells while also inducing apoptosis.

## 1 Introduction

Renal cell carcinoma (RCC) is a prevalent form of tumor in the urinary tract that comprises approximately 3% of the malignant tumors in adults [1]. RCC is the second most common urological tumor. Despite having a lower occurrence than prostate cancer (PCa), RCC exhibits the undesirable fatality rate among malignant neoplasm diseases that impact the urinary system and the prognosis of RCC patients is more severe than that of PCa patients and bladder cancer patients [2,3]. In view of this, it is urgent to find more effective targeted therapies for RCC.

In recent years, the androgen receptor (AR) has become a crucial focus for the cancer therapy. AR is a crucial focus not just in urological cancers, but also in the management of various other types of tumors [4–6]. Most primary and metastatic PCa tumors show AR expression, which is closely linked to PCa advancement [7,8] and tumorigenesis [9]. Due to the grim prognosis and survival rate of RCC patients [10,11], there is an imperative necessity to explore innovative diagnostic and therapeutic approaches for these patients. Research on immune-targeted strategies acquires growing focus in the RCC diagnosis and treatment [12–15]. Traditional Chinese medicines (TCM) offer more cost-effective advantages and less adverse reactions compared to conventional clinical medications. The TCM has the ability to both eliminate tumor cells and prevent cancer by boosting the immune system and health conditions of patients [16–18]. Latterly, the compatibility between TCM and clinical anticancer drugs has been recognized, leading to more effective cancer cell killing and improvements in clinical cancer diagnosis, treatment, postoperative recovery, and prognosis [19–21]. Meanwhile, we found that the lathyrol exhibits tumor suppressive activity against a variety of tumor types by acting on multiple target proteins and several mechanisms. This finding provides a more effective and feasible alternative treatment option for patients with tumors that cannot be treated via surgery operations. Currently, whether lathyrol can affect the biological function of human RCC cells-786-O via affecting AR-related proteins expression is unknown and has not been researched. Hence, we investigated the influences of lathyrol on the malignant activity of 786-O cells through the modulation of AR and prostate-specific antigen (PSA) expression, thereby revealing the antitumor mechanism of lathyrol.

## 2 Materials and methods

### 2.1 Major materials

The following reagents and materials were used in this study: 786-O human RCC cell line authenticated by RTS (Short Tandem Repeat) authentication and mycoplasma tests (Procell Life Science & Technology Co., CL-0010); lathyrol (Weikeqi-Biotech, Sichuan, China, Wakq-00424); nilutamide (AR blocker; Abmole Biotechnology Co., Ltd); fetal bovine serum (FBS; Absin Bioscience); CCK-8 reagent and phosphate buffer saline (PBS) buffer (Biosharp); 1640 basal medium, trypsin-ethylene diamine tetraacetic acid (0.25%) digestion solution, cell culture-grade dimethylsulfoxide (DMSO), tissue and cell radio-immunoprecipitation assay (RIPA) lysis buffer, phenylmethanesulfonyl fluoride, goat serum, hydrogen peroxide (H_2_O_2_), Mayer’s hematoxylin staining solution, crystal violet staining solution, and neutral balsam (Solarbio); enhanced chemiluminescence (ECL) developing solution, horse radish peroxidase (HRP)-labeled sheep anti-rabbit secondary antibody for immunohistochemistry, and diaminobenzidine (DAB) chromogenic kit (Servicebio); bicinchoninic acid assay (BCA) protein quantification kit, terminal deoxynucleotidyl transferase-mediated dUTP nick end labeling (TUNEL) cell apoptosis detection kit (green fluorescence), Triton X-100 (cell permeabilization agent), 4′,6-diamidino-2-phenylindole nuclear stain, mounting medium with anti-fluorescence quenching reagent, and HRP-labeled goat anti-rabbit secondary antibody (Beyotime Biotechnology); 6-well Transwell plates and Matrigel (Corning Biotechnology); rabbit polyclonal anti-glyceraldehyde-3-phosphate dehydrogenase (GAPDH) antibody (internal control; Hangzhou Xianzhi Biotechnology); rabbit polyclonal anti-Ki67 antibody (Bioss Biotechnology); 4% paraformaldehyde fixative solution and anti-phosphorylated adrenergic receptor (p-AR) antibody (phosphorylation site, S650; Absin Biotechnology); protein marker (10–180 kD), anti-phosphorylated Akt (p-Akt) antibody (phosphorylation site, Ser473), rabbit polyclonal anti-Bax antibody, anti-Bcl-2 antibody, anti-matrix metalloproteinase 2 (MMP2) antibody, anti-MMP9 antibody, and anti-cleaved-caspase3 & caspase3 antibody (Wuhan Sanying Biotechnology); rabbit polyclonal anti-AR antibody and rabbit monoclonal anti-cleaved-caspase9 & caspase9 antibody (Affinity Biotechnology); and rabbit monoclonal anti-PSA antibody (STARTER Biotechnology).

### 2.2 Methods

#### 2.2.1 Cell culture

786-O cells were regularly grown at constant-temperature cell incubator (with 37°C, 5% CO_2_, and 95% humidity) in cell culture medium (contained RPMI 1640 medium, 10% FBS, and 1% penicillin and streptomycin). When the cells reached about 85% confluence on the culture flask surface, the cells in the logarithmic phase of growth were digested and utilized in the subsequent experiments.

#### 2.2.2 Experimental grouping

**Figure 1:**
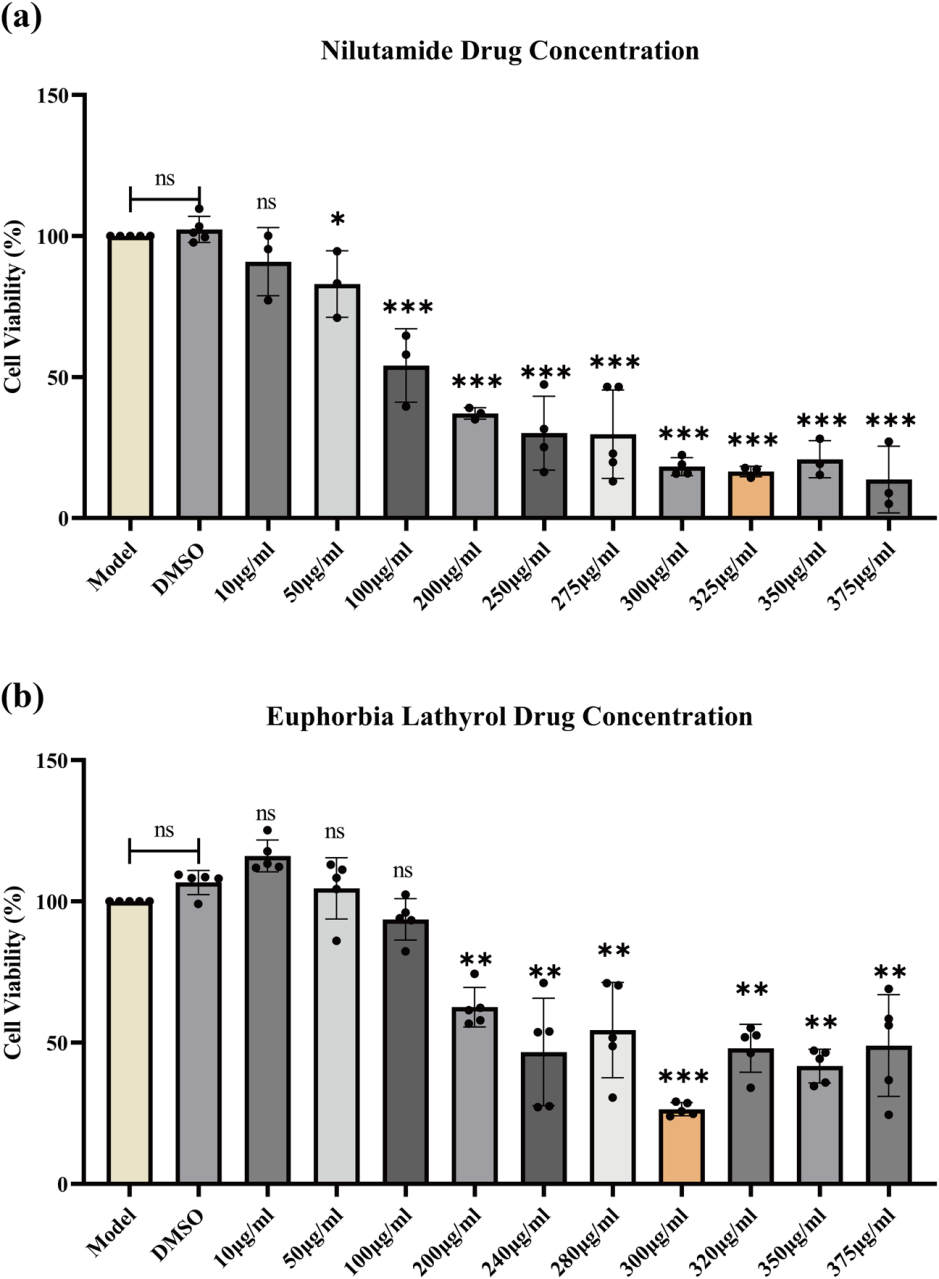
CCK-8 assay was utilized to figure out the diverse drug concentrations used for subsequent experiments. (a) Concentrations of lathyrol used for subsequent experiments and (b) nilutamide concentrations used for the same purpose. The model group cells, which were not treated, were cultured in cell culture medium. For the remaining groups, the drugs were added to the medium at specific concentrations such that the DMSO level did not exceed 1%. The results obtained for the model group compete with the DMSO control group, and the results obtained for the remaining drug concentrations treatment group were compared with A group. According to above CCK8 results, the concentration gradient function was established, and the IC75, IC50, and IC25 values of lathyrol against 786-O cells were 340.86, 293.72, and 202.66 μg/mL. The IC75, IC50, and IC25 values of nilutamide against 786-O cells were 282.54, 293.72, and 47.10 μg/mL.

First, we added 1% DMSO to the medium and performed a contrast experiment. The results showed that the effect of DMSO on the cell viability of 786-O cells cultured in normal medium was not significantly different from that of 786-O cells cultured in normal medium. Therefore, we used this group as the DMSO control group. Moreover, an experimental plan was developed to precisely determine the minimum drug concentration required for nilutamide and lathyrol to exert the maximum anticancer effect, and this concentration was used as the standard concentration for subsequent experiments to further explore the anticancer efficacy of these two drugs. Therefore, based on the results of preliminary experiments (Figure 1a and b), 786-O cells were divided into three groups at random: the DMSO control group (A group), negative control group (B group), and experimental group (C group), and the appropriate drug concentration for each group was determined. Cells in A group were grown in DMSO working medium (contained RPMI 1640 medium and 1% DMSO), B group cells were cultured in nilutamide working medium (contained DMSO working medium and corresponding nilutamide drug concentrations), while those in C group were cultured in lathyrol working medium (contained DMSO working medium and corresponding lathyrol drug concentrations).

#### 2.2.3 Assessment of cell proliferation ability by the CCK8 assay

786-O cells in the exponential phase of growth were plated in 96-well culture plates at a concentration of 8 × 10^3^ cells/mL (100 μL per well). After cell attachment, 100 μL of 1640 medium with the appropriate drug concentration was added and cultured for 24 h. At specified intervals, 10 μL of CCK-8 reagent was uniformly mixed into the medium in each well, and the cells were further incubated at 37°C for an additional 3.5 h in an incubator saturated with 5% CO_2_. Using a microplate reader to measure the absorbance at 450 nm, the culture medium served as a blank control for determining cell viability. The percentage of cell viability (%) = [(AS − Ab)/(Ac − Ab)] × 100%.

#### 2.2.4 TUNEL staining detect cell apoptosis

The cells were immersed in 75% alcohol for 20 min, washed three times with 1× PBS, and placed in 6-well plates, with three replicates for each group. 786-O cells were placed in 6-well culture plates at a seeding density of 1 × 10^5^ cells/mL. Three replicates of each group were included. Once the cells had adhered and reached 80% confluence in the 6-well culture dishes, the media was supplemented with RPMI 1640 medium containing the appropriate drugs for 24 h. 786-O cell apoptosis was evaluated strictly following the guidelines provided with the TUNEL apoptosis detection kit. Nuclei and/or cell debris of apoptotic cells emitted green fluorescence, and nuclei of nonapoptotic cells emitted blue fluorescence. The changes in the fluorescence of 786-O cell nuclei were observed under a fluorescence microscope. The average value was calculated by randomly selecting three high-power fields for each group. The apoptosis fluorescence intensity (AFI) was quantitatively analyzed using ImageJ as follows: AFI = (area of apoptosis green fluorescence in the field of view/area of the nucleus in the field of view).

#### 2.2.5 Scratch assay for the assessment of the cell migration ability

786-O were plated in 6-well culture plates at a density of 1 × 10^5^ cells/mL (with three replicates of each group). Once the cells had adhered and reached 90% confluence in the 6-well culture plate, the old 1640 medium was replaced with fresh medium, and the cells were incubated for 12 h. A sterile 10 μL pipette tip was used to vertically scratch the cell monolayer, after which the medium was removed, and the scratched cells were washed with 1× PBS. 1640 medium with a suitable drug concentration was added to each well. The cells were placed in an incubator with a temperature of 37°C and 5% CO_2_ for a duration of 24 h. Subsequently, the width of the scratch was carefully examined and captured using a high-powered magnifying lens. Cell migration index (%) was calculated at follows: cell migration distance at 24 h/cell migration distance at 0 h × 100%.

#### 2.2.6 Detection of cell invasion ability using a Transwell chamber

The same drug treatment was applied to the cells as described for the scratch assay. After treatment for 24 h, the cells were digested via trypsin and subsequently transferred to Transwell plates. Prior to cell addition, the 6-well plate and Transwell chamber were immersed in 1× PBS for 5 min to moisten the chamber. Subsequently, 80 µL of Matrigel was introduced into the chamber, and the chamber was incubated at 37°C for 30 min to allow the gel to solidify. 786-O cells were subsequently diluted in serum-free 1640 medium to a concentration of 4 × 10^4^ cells/mL. For each group, triplicate wells were established by adding 2 mL cell culture medium (contained RPMI 1640 medium and 10% FBS) to the lower chamber after cell seeding (1 mL of cell suspension) in the upper chamber. The cells were incubated at 37°C for 24 h. After the 24 h culture period, the cells located on the upper chamber surface (specifically, cells that had not penetrated into the lower chamber) were delicately eliminated using a cotton swab. After fixation in 4% paraformaldehyde for 15 min, the cells that had entered the lower chamber were stained with 0.25% crystal violet for 30 min and then observed using a microscope. ImageJ software was used to count the number of cells that invaded the lower chamber in randomly selected low-power and high-power fields, which included the central and peripheral membranes.

#### 2.2.7 Western blot analysis of the expression of Ki67, Bcl-2, Bax, caspase9, cleaved-caspase9, caspase3, cleaved-caspase3, AR, p-AR, and p-Akt in cells

After 24 h of the same treatments used for the scratch assay, the cells were enzymatically digested with trypsin and subsequently transferred to a 1.5 mL centrifuge tube. The tube was then centrifuged at a speed of 12,000 rpm for 10 min. Proteins were extracted by lysing the cells in ice-cold RIPA lysis buffer. BCA assay was used to determine the concentration of the proteins. Following SDS‒PAGE, the proteins were transferred to polyvinylidene fluoride membranes, and the membranes were subsequently blocked using tris buffered saline tween (TBST) (including 5% skim milk powder) and incubated overnight at 4°C on a shaker with primary antibodies diluted in TBST (including 5% skim milk powder). The dilutions used for the AR, proliferation and apoptosis related protein antibodies are shown in Table 1. Afterward, the membranes were placed on a shaker and incubated with TBST (containing 5% skim milk powder) diluted with HRP-labeled secondary antibodies at room temperature for 2.5 h. Subsequently, the membranes were incubated with ECL reagent. The bands were scanned, and the gray values of the bands were quantitatively analyzed by ImageJ software.

**Table 1:** Primary antibody dilutions for WB

Primary antibody	Dilution ratio
AR	1:1,000
p-AR	1:1000
Ki67	1:1,000
Bax	1:5,000
Bcl-2	1:5000
p-Akt	1:5,000
Caspase3	1:1,000
Caspase9	1:1,000
GAPDH (internal reference)	1:1,000

#### 2.2.8 Evaluation of the expression of MMP2, MMP9, and PSA in cells by immunocytochemistry (ICC)

Coverslips (20 mm) were soaked in 75% alcohol for 20 min, washed three times with 1× PBS, and placed in 6-well plates. The cells were distributed at a concentration of 1 × 10^5^ cells/mL in 6-well culture plates, with three replicates for each group. The treatments used were the same as those used in the scratch assay. After 24 h, the medium containing the drug was removed, and the cells were rinsed twice with 1× PBS and then immersed in 1 mL of 4% paraformaldehyde for 15 min. Subsequently, the coverslips were carefully placed onto glass slides using tweezers. Following permeabilization using %Triton X-100 (diluted in 1× PBS) for 5 min at ambient temperature, 3% H_2_O_2_ was gradually introduced into the cells to inhibit their natural peroxidase activity. Subsequently, the cells were maintained at room temperature for 30 min. To block nonspecific binding, goat serum working solution was subsequently added dropwise to the cells, and the cells were then incubated for 30 min at room temperature. After dilution of the primary antibodies (the dilution ratios are shown in Table 2), the cells were incubated in a wet box at 4°C overnight, and the antibodies were then added dropwise. The cells were subsequently subjected to three 3 min washes with PBS and then dried with absorbent paper. Next, a goat anti-rabbit secondary antibody conjugated to HRP (at a dilution ratio of 1:300) was added, and the samples were incubated at room temperature for 30 min. Freshly prepared DAB chromogenic solution was added dropwise, and nuclear counterstaining was performed with Mayer hematoxylin. The slides were mounted using neutral balsam, and the average optical density (AOD) was quantified using ImageJ software.

**Table 2:** Primary antibody dilutions for immunocytochemistry

Primary antibody	Dilution ratio
MMP2	1:100
MMP9	1:100
PSA	1:200

### 2.3 Statistical analysis

Cells are divided into three groups. A group is the DMSO control group, B group is the negative control group, and C group is the experimental group. The data were analyzed using IBM SPSS 26.0, and the figures were generated using GraphPad Prism 9.5. All cell experiments were proceeded with triplicated independent biological replicates. A difference was considered statistically significant if *P* < 0.05, *P < 0.05, **P < 0.01, or ***P < 0.001; alternatively, a difference is considered not significant if *P* > 0.05.

## 3 Results

### 3.1 Lathyrol inhibited the proliferation of 786-O human RCC cells

**Figure 2:**
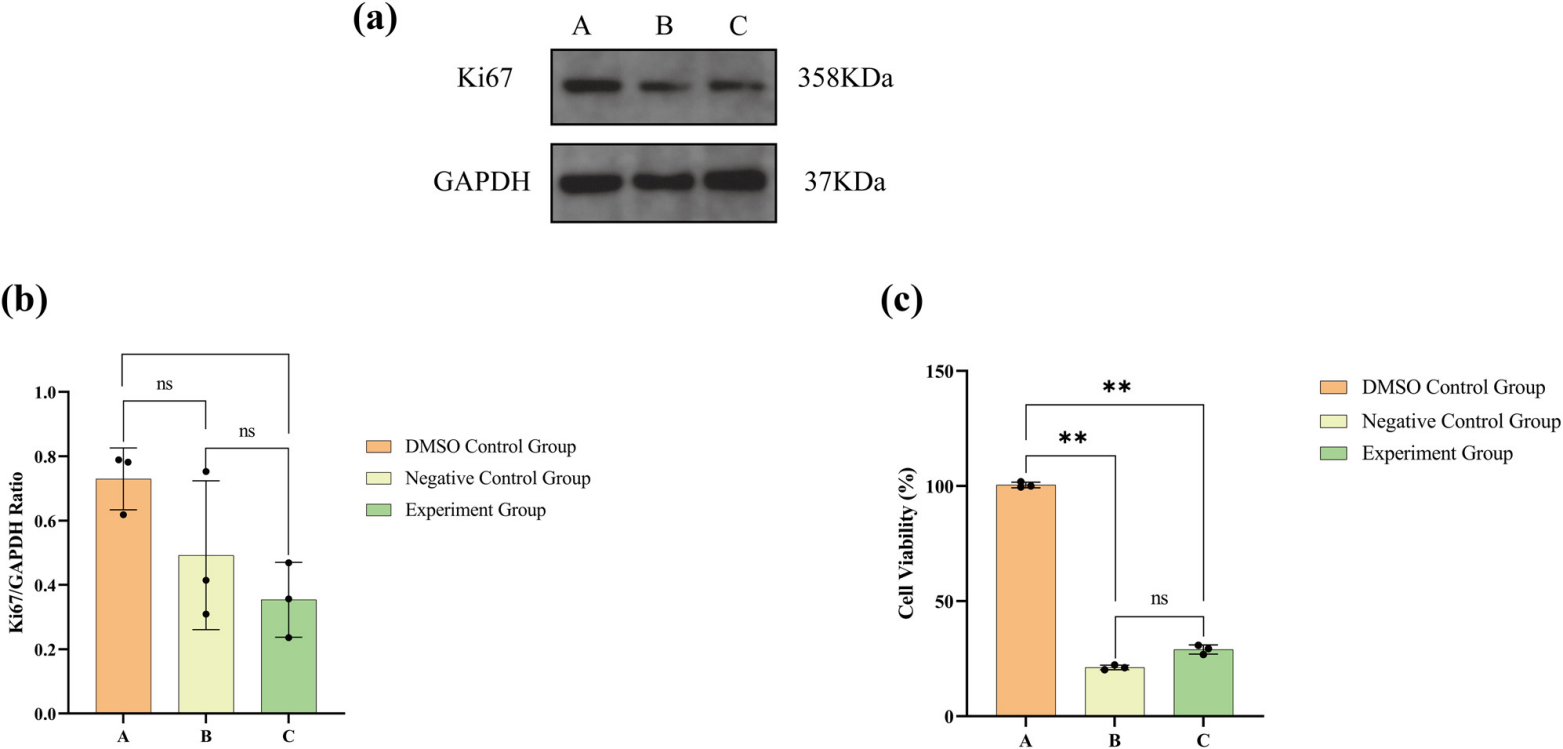
(a) Western blot results for Ki67 and GAPDH protein expression, (b) statistical analysis of Ki67 expression levels, and (c) statistical analysis of the CCK-8 results. The numerical details can be seen in Figure 10a(a) and (b).

After a 24 h treatment period, the viability of 786-O cells in both the B and C groups exhibited a significant reduction (Figure 2c). Additionally, the secretion levels of the Ki67 protein were diminished in comparison to the 786-O cells in group A (Figure 2a). This finding suggests that both lathyrol and nilutamide demonstrate substantial inhibitory effects on the proliferative capacity of RCC 786-O cells. Notably, the proliferative potential of 786-O cells in the negative control, as well as in the experimental B and C groups, was significantly lower than that observed in the DMSO control group A (*P* < 0.05). Furthermore, the expression level of Ki67 in the experimental groups was significantly reduced compared to the other two groups (*P* < 0.05). However, no statistically significant difference was found in Ki67 expression between the negative control and DMSO control groups B and C (*P* > 0.05).

### 3.2 Lathyrol promoted the protein expression of Bcl-2, cleaved-caspase3, and cleaved-caspase9 and inhibited the protein expression of Bax in 786-O cells

**Figure 3:**
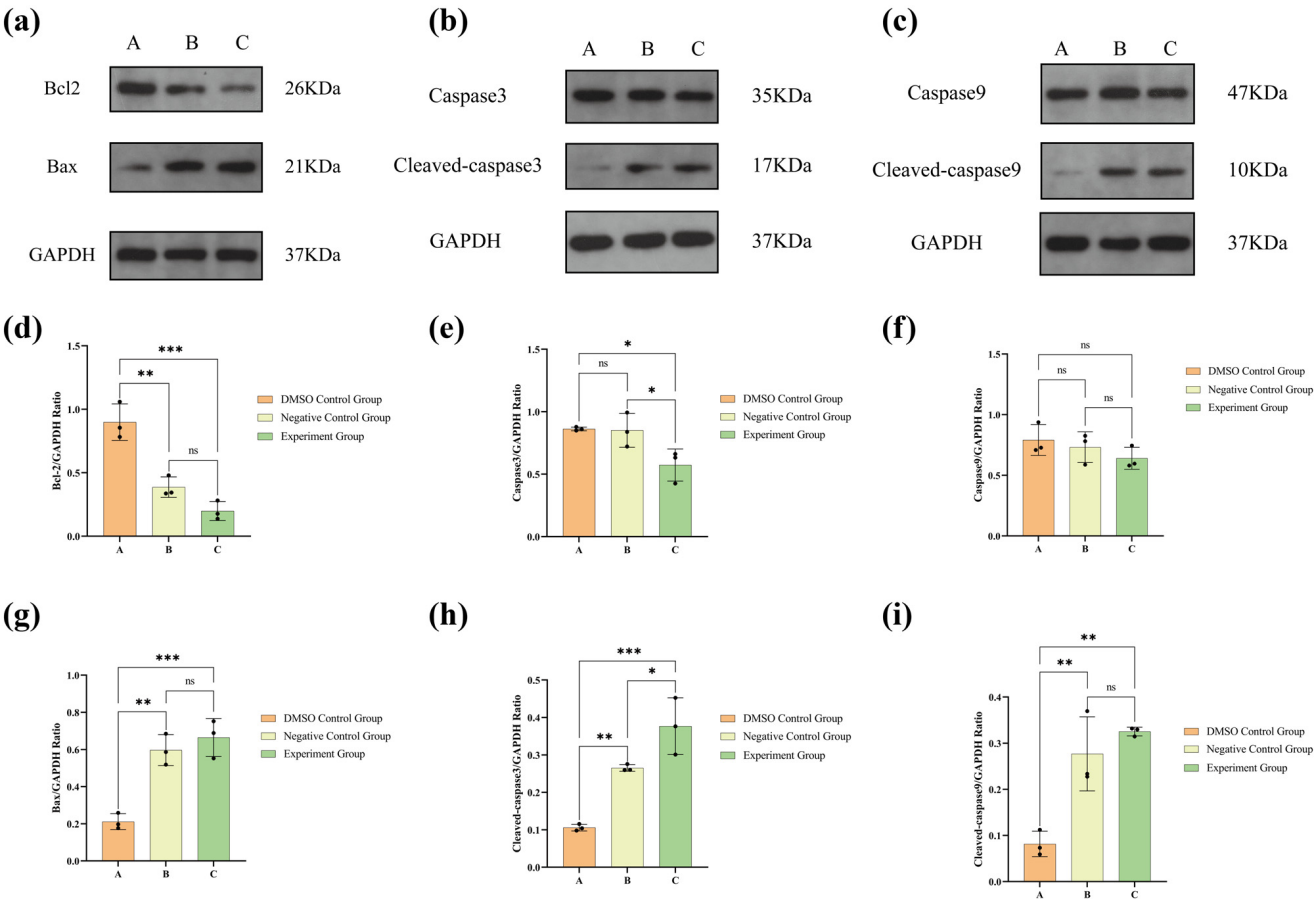
(a) Western blot results are presented for the protein expression of Bcl-2, Bax, and GAPDH. (b) Western blot results for caspase3, cleaved-caspase3, and GAPDH expression. (c) Western blot results for caspase9, cleaved-caspase9, and GAPDH expression. (d) Statistical analysis for Bcl-2 expression. (e) Statistical analysis for caspase3 expression. (f) Statistical analysis for caspase9 expression. (g) Statistical analysis for Bax expression. (h) Statistical analysis for cleaved-caspase3 expression. (i) Statistical analysis for cleaved-caspase9 expression. The numerical details can be seen in Figure 10a(c)–(h).

Under the treatment, the apoptosis of 786-O cells was enhanced. In particular, both lathyrol and nilutamide exhibited a pronounced capacity to significantly facilitate the apoptotic process in 786-O cells. As illustrated in Figure 3, the DMSO control group (Group A) demonstrated elevated levels of Bcl-2 protein expression alongside diminished levels of Bax, cleaved-caspase3, and cleaved-caspase9 protein expression when compared with the control groups B and C. Additionally, the negative control group B and the experimental group C showed considerably increased expression levels of Bax, cleaved-caspase3, and cleaved-caspase9, while also exhibiting reduced Bcl-2 protein expression in comparison to the DMSO group (*P* < 0.05). However, no statistically significant differences were observed in the expression levels of caspase3 and caspase9 among the three groups (*P* > 0.05).

### 3.3 Lathyrol promoted the apoptosis of 786-O cells

**Figure 4:**
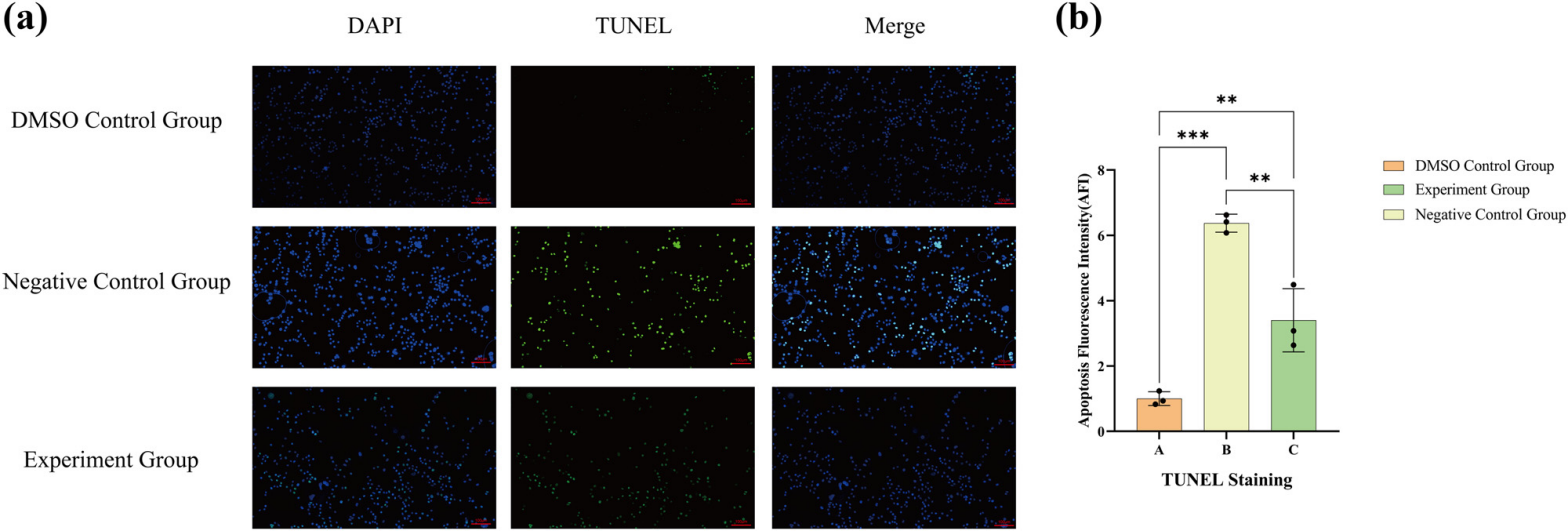
(a) Outcomes of the TUNEL staining of 786-O cells in different groups following medication. (b) Statistical evaluation of the AFIs in each group. The numerical details can be seen in Figure 10b(a).

The TUNEL test revealed that the DNA fragmentation of 786-O cells in groups B and C was more pronounced (Figure 4a). Concurrently, the AFIs of 786-O cells in groups B and C were more significant than those in group A (Figure 4b). Importantly, both lathyrol and nilutamide markedly enhance the apoptosis of 786-O cells. The findings are illustrated in Figure 4. The negative control B group and the experimental C group demonstrated significantly elevated AFIs and a notably higher fluorescence intensity compared to the DMSO control A group (*P* < 0.05).

### 3.4 Lathyrol inhibited the migration of 786-O cells

**Figure 5:**
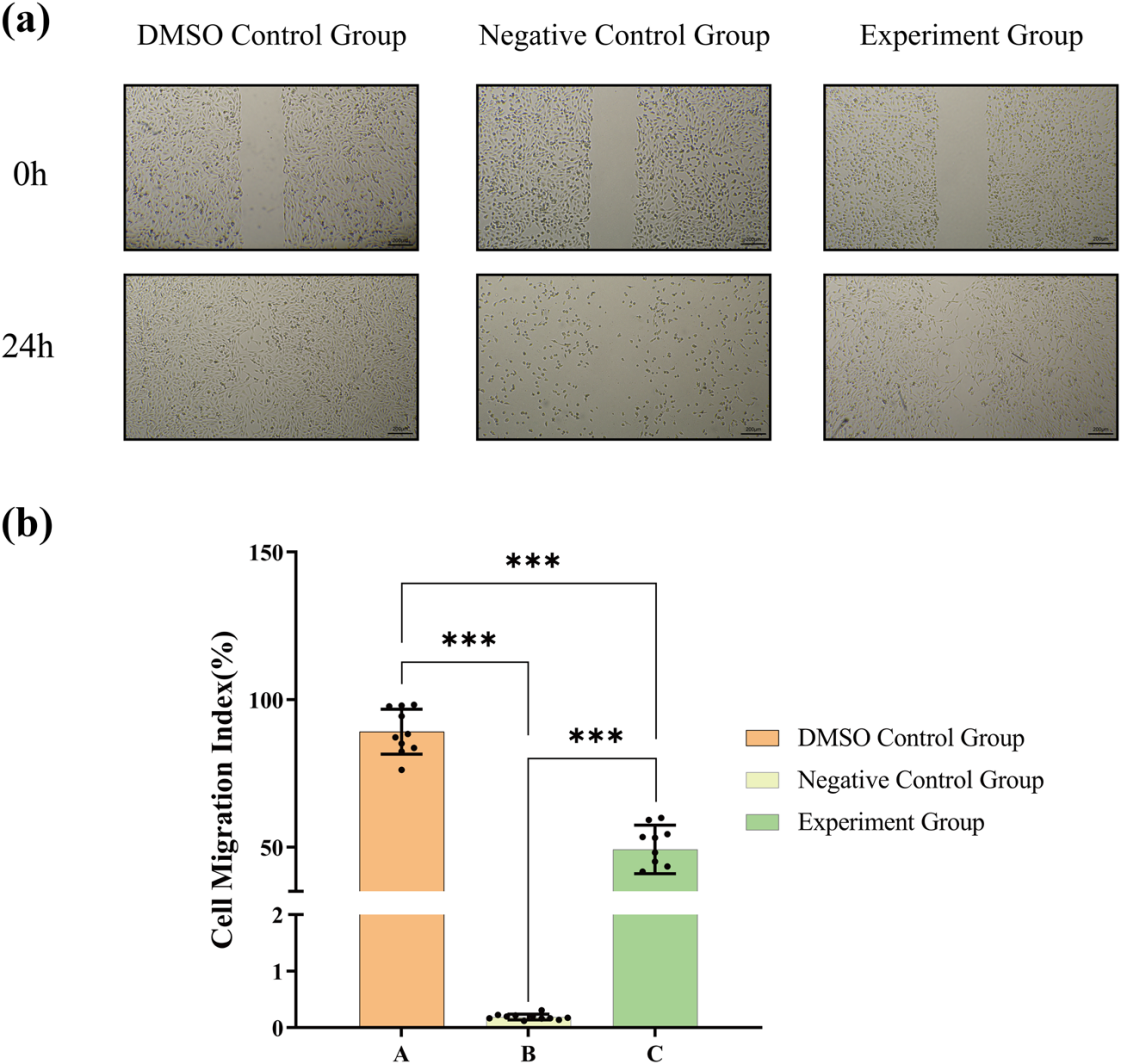
(a) Images of the scratch assay of 786-O cells in each group after drug treatment. (b) Statistical analysis of the cell migration rate of each group. The numerical details can be seen in Figure 10b(b).

The results of scratch experiment showed (Figure 5a) that 786-O cells in group A could almost completely fill the scratch wound, while the migration ability of 786-O cells in groups B and C was significantly inhibited, leaving significant scratch gaps. In addition, a significant inhibitory effect of lathyrol and nilutamide on cell migration ability was also observed. In addition, the cells in the B and C groups had a reduced migration distance and lower cell migration rate compared to A group, with statistical significance (*P* < 0.05).

### 3.5 Lathyrol inhibited the invasion of 786-O cells

**Figure 6:**
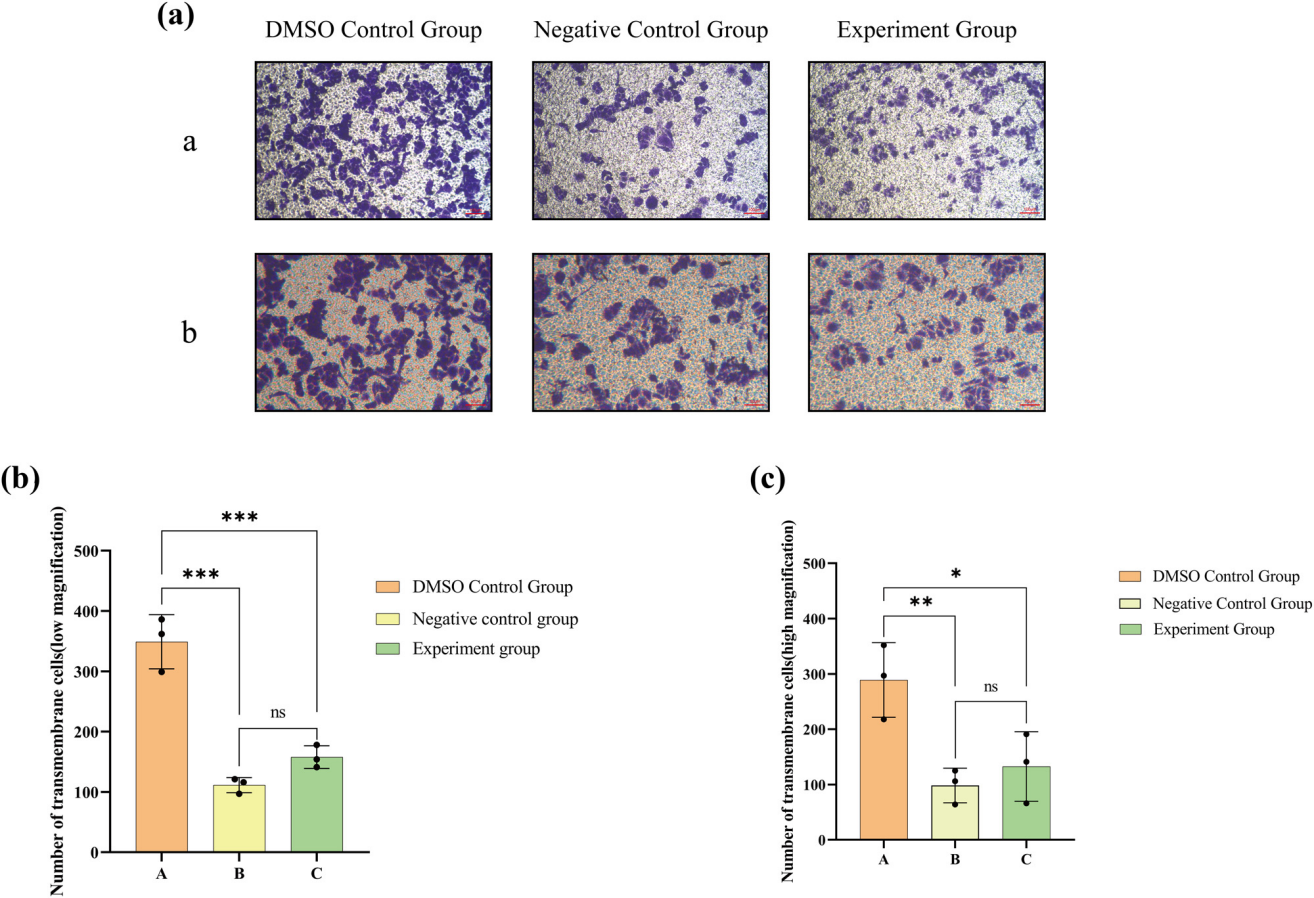
(a) Outcomes of the Transwell tests on 786-O cells in different groups following medication; ((a) depicts the findings of the Transwell invasion test under low magnification (×100); while (b) illustrates the results of the Transwell invasion test under high magnification (×200). Cells that moved across the Transwell membrane were colored in a blue-purple hue using crystal violet staining solution). (b) Statistical analysis of cell migration through the Transwell membrane in each group at low magnification. (c) Statistical analysis of cell migration through the Transwell in each group at high magnification. The numerical details can be seen in Figure 10b(c) and (d).

Transwell experiment results (Figure 6a) demonstrate that at low magnification, the quantity of cells that infiltrated the Matrigel in both the negative control and experimental groups was notably less than 786-O cells in the A group; at high magnification, the number of cells that infiltrated the Matrigel in the A group was meaningfully greater than the other groups (*P* < 0.05). There was no notable statistical distinction observed between the B and C groups. Hence, lathyrol and nilutamide exhibit a marked inhibitory effect on the invasive capacity of 786-O cells.

### 3.6 Lathyrol inhibited the protein expression of MMP2 and MMP9 in 786-O cells

**Figure 7:**
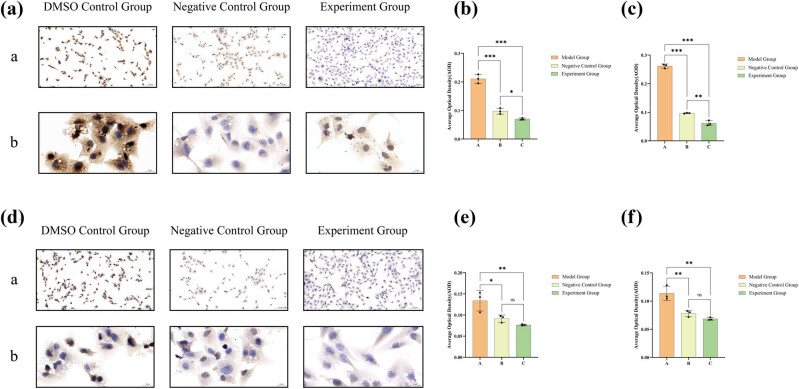
Results of ICC staining for MMP2 expression in (a), and the combined results of ICC and DAB staining for MMP9 expression in (d). The appearance of brown granules in these sections serves as an indicator of positive expression. (a) Displays low-magnification images (×100) for closer inspection, while (b) offers high-magnification images (×400) for a more detailed view. (b) Statistical analysis of AODs pertaining to MMP2 expression at low magnification and (c) analogous analysis for MMP2 expression at high magnification. (e) Statistical analysis of AODs for MMP9 expression under low magnification and (f) corresponding analysis for MMP9 expression at high magnification. The precise numerical details can be found in Figure 10b(e)–(h).

As shown in Figure 7 that MMP2 was mainly expressed in the cytoplasm of 786-O cells, but its presence was also detected in the nucleus (Figure 7a), and MMP9 localized mainly to the cell membrane (Figure 7b). In general, 786-O cells in groups B and C showed a significant inhibitory trend in the protein expression of MMP2 and MMP9 compared to other groups (Figure 7a and b). At low magnification (Figure 7a(a) and d(a)), the levels of MMP2 and MMP9 expression in both B and C groups were notably reduced compared to A group. At high magnification (Figure 7a(b) and d(b)), the levels of MMP2 and MMP9 in the A group were notably elevated compared to the B and C groups (*P* < 0.05). Due to that, lathyrol and nilutamide exhibit a marked inhibitory effect on the invasive capacity of 786-O cells.

### 3.7 Lathyrol promoted the expression of the AR, p-AR, and PSA proteins and inhibited the expression of the p-Akt protein in 786-O cells

**Figure 8:**
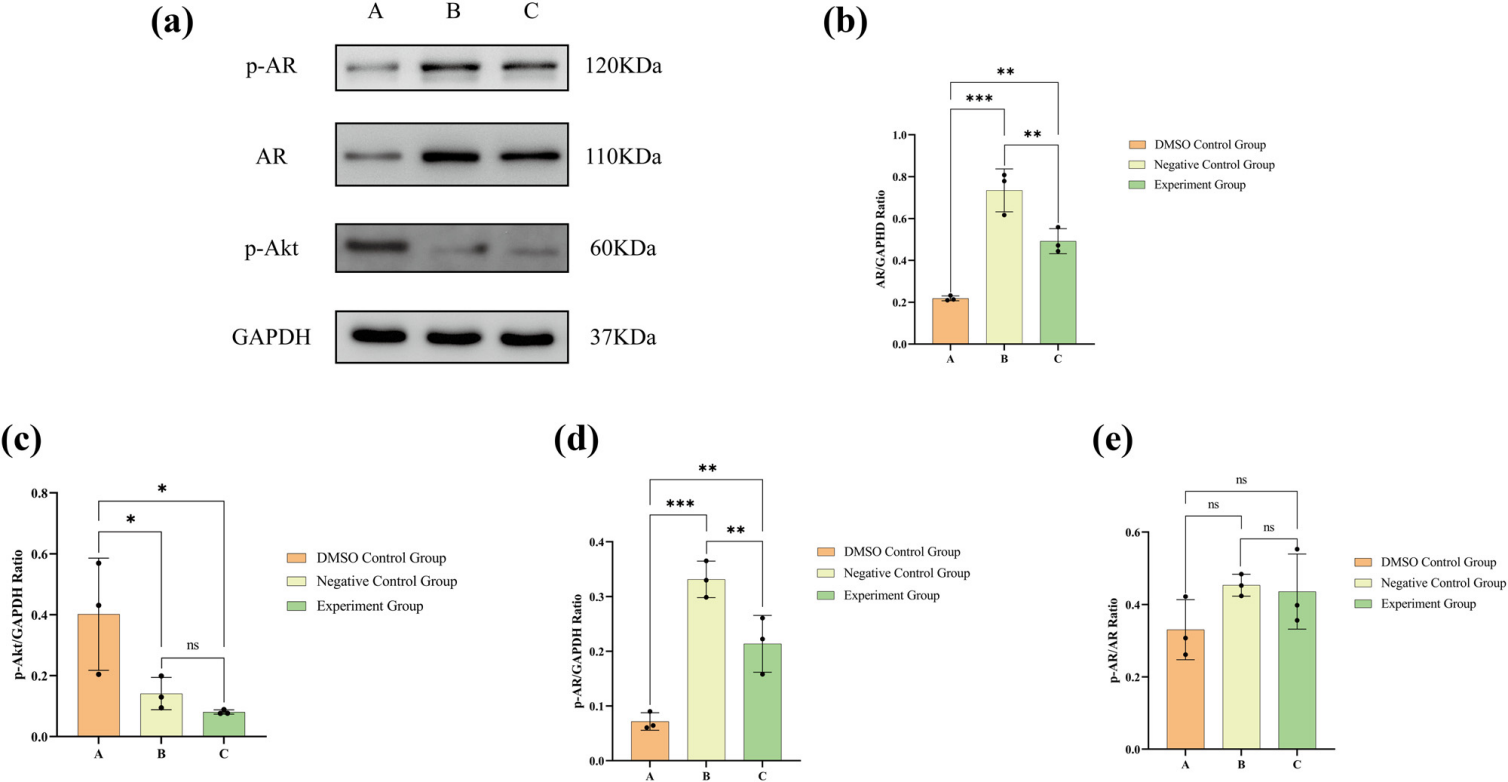
(a) Western blot outcomes for the protein expression levels of p-AR, AR, p-Akt, and GAPDH; (b) statistical evaluation of AR expression; (c) statistical evaluation of p-Akt expression; (d) statistical evaluation of p-AR expression; and (e) statistical evaluation of the p-AR/AR ratio. The numerical details can be seen in Figure 10c(a)–(d).

**Figure 9:**
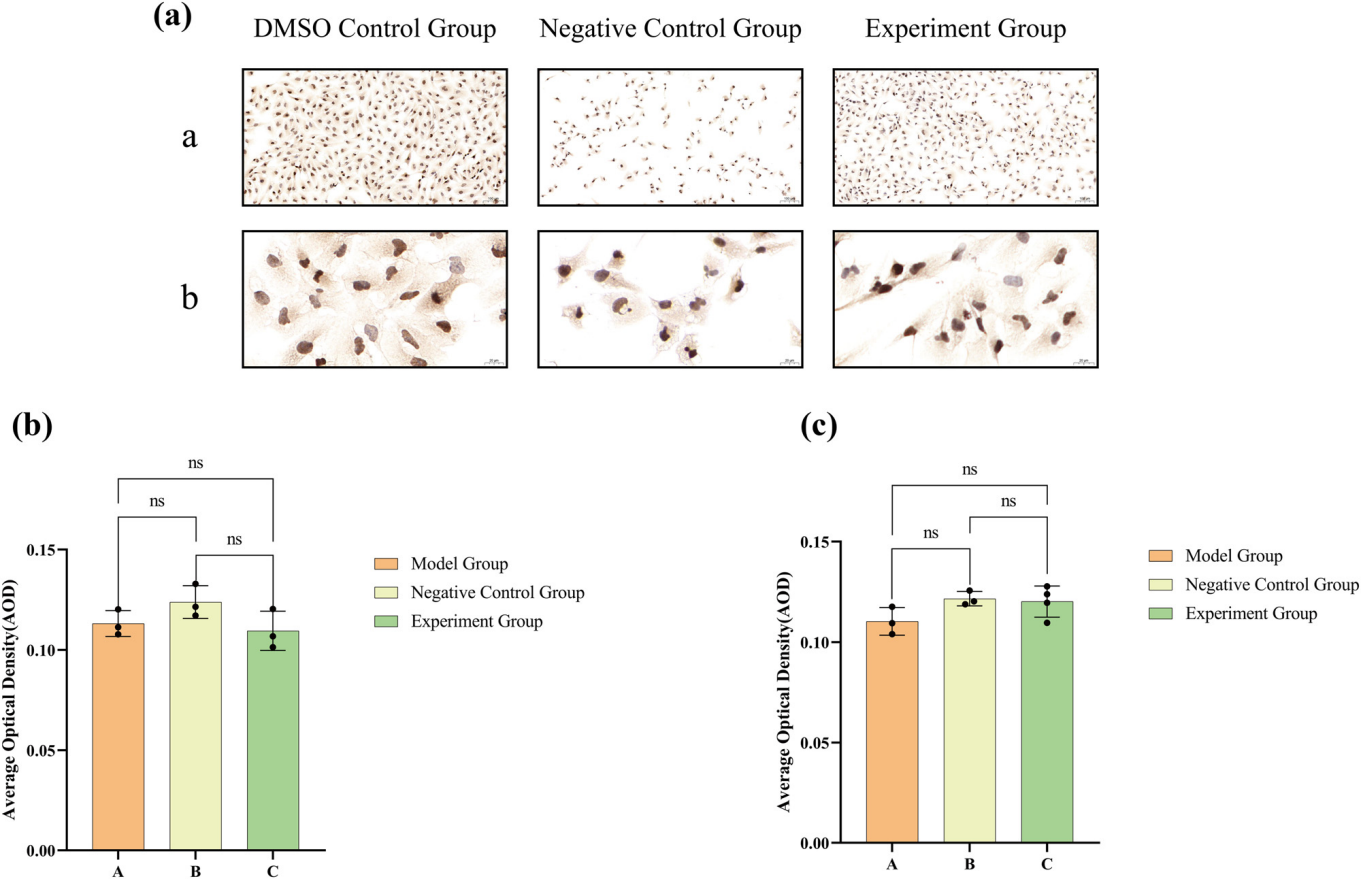
ICC staining and DAB color development results for PSA expression are displayed in (a), with brown granules indicating positive expression. ((a) includes low-magnification (×100) images, while (b) includes high-magnification (×400) images). The statistical analysis of the AOD data for PSA expression under low magnification is shown in (b), and under high magnification in (c). The numerical details can be seen in Figure 10c(e) and (f).

After drug administration, in comparison to the A group’s 786-O cells, the B and C groups manifested an upregulation of AR and p-AR protein expression, accompanied by a downregulation of p-AR expression (Figure 8a). The 786-O cells within both B and C groups show markedly increased levels of AR and p-AR, accompanied by a notably diminished level of p-Akt, in comparison to the 786-O cells in A group. Nonetheless, there was no notable variation in the p-AR/AR ratio observed across the three groups (*P* > 0.05). Moreover, ICC results (Figure 9a) displayed that PSA was expressed mainly in the nucleus and the results of PSA expression did not exhibit any statistically significant difference (*P* > 0.05) among the three groups, as observed under both low and high magnification conditions (Figure 9a(a) and (b)).

**Figure 10:**
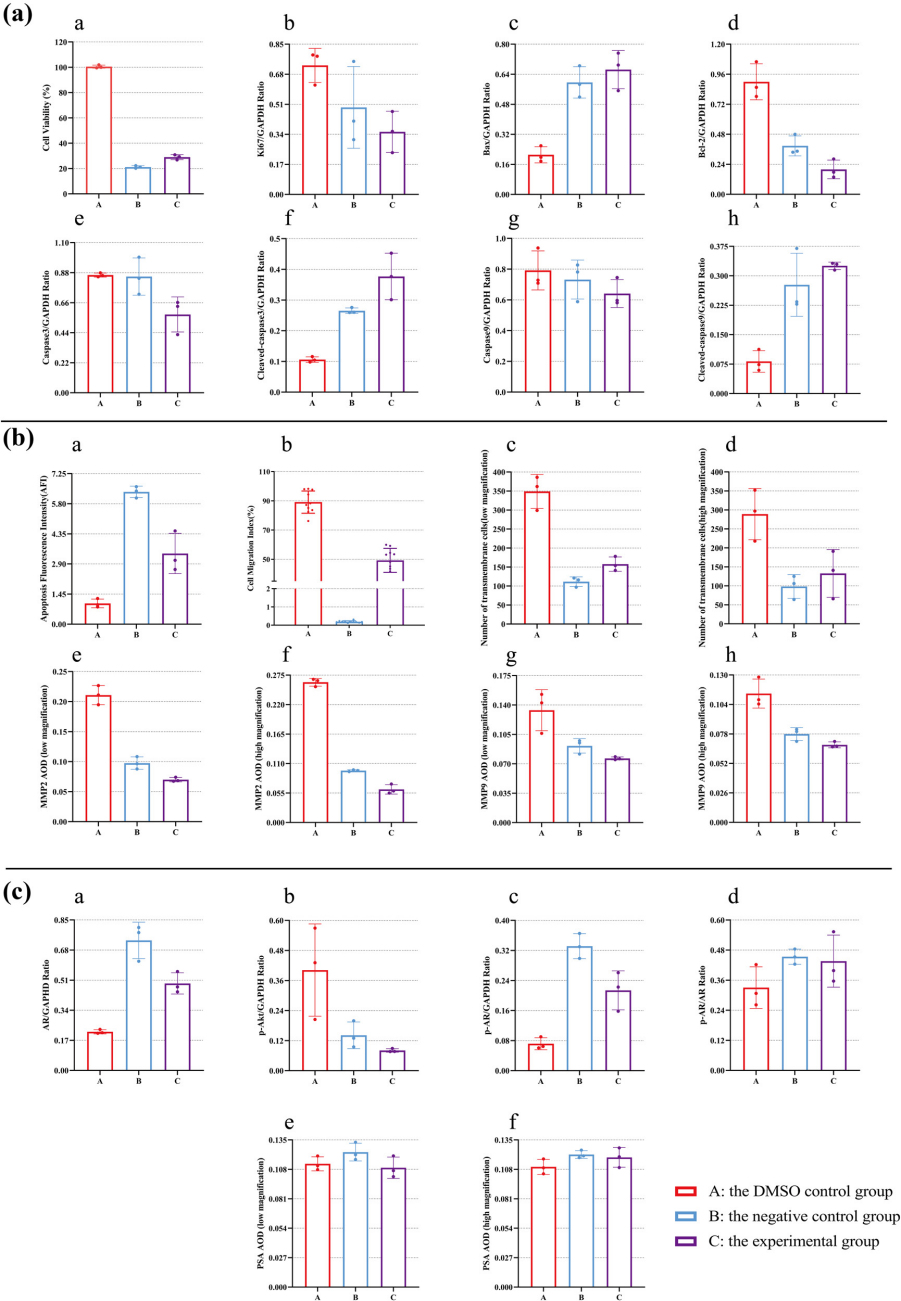
Summary of numerical results. This figure is a summary and visualization of the numerical results from Figures 2–9. The specific experimental results can be found in the corresponding experimental results’ panel.

## 4 Discussion

Initial signs of RCC in patients are not typical. However, those in advanced stages may have symptoms such as painless blood in urine, lower back pain, a mass in the abdomen, and other signs. The initial signs of RCC may not be readily apparent, and approximately 30–40% of individuals diagnosed with RCC already exhibit metastasis. Nevertheless, around 30% of individuals with RCC who receive surgery will encounter a relapse, potentially leading to the development of metastatic RCC (mRCC) [22]. Patients with mRCC have a grim prognosis, as their 5-year survival rate is below 10% and their average survival is less than 1 year [3,11,23]. Radical nephrectomy continues to be the primary treatment for RCC in clinical practice [24,25]. Patients who receive early surgical intervention have a more favorable prognosis than patients who do not. However, advanced RCC does not respond well to radiotherapy or chemotherapy [9]. Surgical treatment can only alleviate symptoms and reduce the tumor size, limiting RCC patients to opting for palliative care [26–28]. Therefore, effective treatment options for RCC urgently need to be developed.

The traditional Chinese herbal medicine system in China has high economic benefits and has demonstrated notable clinical effectiveness against tumors, with reduced side effects and toxicity [29,30]. As a TCM belonging to the genus Euphorbia, *Leptochloa chinensis*, which is warm in nature and pungent in taste has low toxicity, regulates the large intestine, liver and kidney, removes water, reduces swelling and blood stasis to eliminate symptoms, and exerts therapeutic effects on diseases such as phlegm retention, stagnation of swelling and edema. In recent years, studies of the antitumor activity of *L. chinensis* have led to further developments in the study of natural medicines for the treatment of tumors. Lathyrol is the dry mature seed of *Euphorbia lathyris* L., a TCM that are effective against tumors and can directly eliminate cancer cells, suppress the growth of tumor tissue, control the tumor microenvironment (TME), and reduce tumor cell resistance to drugs, ultimately enhancing the effectiveness of cancer treatments [31–33].

According to recent research, the activation and expression of AR can stimulate the growth and advancement of tumors at later stages. Moreover, the activation and expression of AR can promote the invasion, dissemination, growth, and advancement of cancer cells through different pathways, such as inhibiting the release of immune factors, boosting tumor immune escape, stimulating angiogenesis, and triggering the EMT process. AR also being utilized in the treatment of RCC, is involved in malignant biological behaviors, such as drug resistance [34–40], and is a well-known immune target. PSA is essential for the advancement of PCa cells as it serves as a target gene of AR and plays a significant role in the AR signaling pathway [41]. PSA levels rise as PCa advances, but inhibiting PSA can slow the proliferation and metastasis of cancer cells [42]. Phosphorylated serine/threonine protein kinase (p-Akt) is a key molecule in the AR signaling pathway and an important molecular target of AR signaling that combines with other pathways to exert its effects. Inhibiting the proliferation and migration ability of cancer cells and enhancing the effectiveness of clinical anticancer medications can be achieved by reducing the p-Akt/Akt ratio [43,44].

In pilot experiments, we investigated the anticancer effects of lathyrol and nilutamide by determining the most effective concentrations and revealed the anticancer mechanism of lathyrol and nilutamide against RCC cells. According to the ICC data, lathyrol and nilutamide enhanced the expression of AR and p-AR while suppressing the expression of p-Akt in 786-O cells. However, the impact of these antagonists on PSA expression was minimal, and to date, the association between lathyrol and AR has not been studied in China or abroad; furthermore, the impacts of AR antagonists on AR remain unclear. Through a literature review and preliminary experiments, we found that human RCC 786-O cells are negative for AR [45,46]. However, the application of AR antagonists may promote the expression of AR in cancer cells. Olson et al. [47] reported that the use of androgen deprivation therapy could enhance the levels of AR in human and mouse PCa cells, both *in vitro* and *in vivo*. Serçinoğlu et al. [48] reported that anti-AR therapy may increase the number of point mutations in AR or affect AR expression. Hence, considering the outcomes of these experiments, we speculate that lathyrol and nilutamide may not exert their anticancer effects through impacting the production of AR. Instead, these compounds likely competitively hinder AR expression, obstruct AR signal transmission, suppress the growth, movement and infiltration of 786-O cells, and stimulate cell apoptosis. Consequently, these effects could lead to an increase in AR variants or feedback stimulation of AR synthesis. It is also possible that while lathyrol and nilutamide exert their anticancer effects, they may promote the transcription and expression of certain factors and further promote AR expression and phosphorylation. Due to that, we believe that nilutamide still has some significance in this study based on its mechanism. As an AR blocker, nilutamide can compete with AR to inhibit its binding to androgen and suppress AR signal transduction, thereby exerting anticancer effects. Because of that the expression of AR in 786-O cells is increased after nilutamide therapy, but the expression of its downstream effector protein PSA is not affected, while the expression of p-Akt is lowered, and the malignant behavior of RCC cells is inhibited. Therefore, we hypothesize that nilutamide inhibits the binding of androgen to AR and inhibits AR signal transduction, thereby inhibiting the proliferation and invasive ability of RCC 786-O cells. Regarding the proliferative characteristics of 786-O cells, both lathyrol and nilutamide effectively suppressed cell proliferation, and lathyrol also suppressed the expression of the proliferation marker protein Ki67. However, nilutamide had a minimal impact on Ki67 protein expression, suggesting that this compound may regulate the proliferation of 786-O cells through alternative mechanisms. Regarding the apoptotic phenotype, the quantity of 3′-OH DNA fragments in 786-O cells was notably higher in both the negative control and experimental groups than in the DMSO group. The severity of the DNA breaks in these two groups was more pronounced than that in the other two groups, indicating that lathyrol and nilutamide could effectively trigger 786-O cell apoptosis. The decrease in the Bcl-2/Bax ratio induced by lathyrol and nilutamide enhanced the expression of cleaved-caspase3 and cleaved-caspase9. The protein expression of caspase3 and caspase9 was minimally affected by lathyrol and nilutamide, whereas the Bcl-2/Bax ratio was reduced. Increased caspase3, caspace9, cleaved-caspase3, and cleaved-caspace9 expression often indicates the occurrence of cell apoptosis [49–51]. Thus, we hypothesized that lathyrol and nilutamide enhance the activity of caspase family members by stimulating the upregulation of caspase3 and inducing the splicing of caspase9. Chen et al. [31] found that lathyrol inhibited the proliferation of A549 and H460 lung cancer cells and promoted the apoptosis of A549 and H460 lung cancer cells. This research provide evidence for our hypothesis. The outcomes from the scratch test demonstrated that the degree of migration of 786-O cells in both the negative control and experimental groups was significantly less than that observed in the model group. Both lathyrol and nilutamide exhibited a substantial inhibitory effect on the motility of 786-O cells, with nilutamide’s effect being notably more pronounced compared to that of lathyrol. Furthermore, Transwell assays, along with the evaluation of MMP expression, serve as crucial techniques for determining the invasive capabilities of cells. MMPs enhance the infiltration and invasion of tumors in the TME by breaking down the extracellular matrix, ultimately facilitating the migration and invasion of tumor cells [52–55]. Following the administration of lathyrol and nilutamide, the number of cells that traversed the Matrigel in both the negative control and experimental groups was significantly lower than that found in the model group. The suppressive impact of nilutamide on 786-O cells was evident. ICC results data show that MMP2 and MMP9 expression levels were reduced in 786-O cells in both the negative control and experimental groups than in the model group, indicating that the inhibitory effect of lathyrol was more pronounced. Hence, the invasion of 786-O cells can be strongly suppressed by lathyrol and nilutamide, consistent with the findings reported by Yan et al. [32]. These authors employed lathyrol to hinder the movement and infiltration of A549 and H1299 cells, which are associated with non-small cell lung cancer. During this process, the AR function of the cells is inhibited, leading to an obvious increase in the expression of AR and p-AR in B and C group cells to be responsive to the anticancer effects of nilutamide and lathyrol. This may be the probable cause of the phenomenon that the proliferation and invasion ability of RCC 786-O cells are inhibited, apoptosis is significantly induced, and AR expression is abnormally elevated.

The functioning of *L. chinensis*, an ancient Chinese remedy, on 786-O human RCC cells and Renca cells was examined in this study. Recently, studies have demonstrated that *L. chinensis* can impact cancer tissues by blocking the NF-κB pathway and related protein expression, inducing apoptosis in RCC cells, and suppressing TGF-β/Smad pathway protein expression and activation. According to recent research, the proliferation and invasion of RCC can be partially hindered by *L. chinensis*, and this effect is achieved by suppressing the production of invasion-related proteins in RCC xenografts and impeding the progression of EMT through the inhibition of crucial proteins such as AR and SPHK-2 [56–58]. Consequently, lathyrol plays a role in restraining the proliferation and invasion of RCC cells to some extent. Overall, we hypothesize that lathyrol can impact the AR expression and important proteins in the AR signaling pathways, thereby inhibiting some biological functions of 786-O human RCC cells, including significantly inhibiting their proliferation, migration, and invasion. However, the lack of studies on the precise gene-level identification and examination of alternative potential targets of lathyrol has hindered any further exploration of the AR pathway and its associated molecular mechanisms. Therefore, additional research is required to clarify the process and specific objectives associated with the progression of RCC, providing new insights for the clinical detection and management of RCC.

Finally, although this experimental study is limited by funding and other issues it still has many shortcomings, we believe that this study is still of certain importance. First of all, the current clinical treatment is still using radical nephrectomy as the main treatment for patients with RCC, and RCC is not sensitive to radiotherapy and chemotherapy. By studying the mechanism of action of lathyrol on RCC, we can provide additional anti-tumor means for patients with RCC who cannot undergo surgical treatment due to age, physical reasons, and economic conditions. Second, as one of the TCM system member, lathyrol has the advantages of high economic efficiency, significant clinical efficacy, and less side effects and toxicity in anti-tumor efficacy, which can provide effective anti-tumor therapy for patients who cannot perform surgical treatment, and plays a key role in the process of anti-tumor diagnosis and treatment. In addition, exploring the immunotargeted anti-tumor mechanism of TCM lathyrol by modern means adds an additional theoretical basis for the study of the mechanism of lathyrol inhibiting tumor growth, and provides novel ideas for the RCC diagnosis and treatment.

## 5 Conclusion

In summary, lathyrol has significant anticancer effects *in vitro* and may inhibit malignant behaviors, such as proliferation, migration, and invasion, in 786-O cells by affecting the expression of AR, inhibiting AR signal transduction, and inducing apoptosis.
